# Health e‐mavens: identifying active online health information users

**DOI:** 10.1111/hex.12398

**Published:** 2015-08-21

**Authors:** Ye Sun, Miao Liu, Melinda Krakow

**Affiliations:** ^1^ Department of CommunicationUniversity of UtahSalt Lake CityUTUSA

**Keywords:** health e‐maven, health information seeking, health maven, maven, pew internet & american life project

## Abstract

**Background:**

Given the rapid increase of Internet use for effective health communication, it is important for health practitioners to be able to identify and mobilize active users of online health information across various web‐based health intervention programmes. We propose the concept ‘health e‐mavens’ to characterize individuals actively engaged in online health information seeking and sharing activities.

**Objectives:**

This study aimed to address three goals: (i) to test the factor structure of health e‐mavenism, (ii) to assess the reliability and validity of this construct and (iii) to determine what predictors are associated with health e‐mavenism.

**Methods:**

This study was a secondary analysis of nationally representative data from the 2010 Health Tracking Survey. We assessed the factor structure of health e‐mavenism using confirmatory factor analysis and examined socio‐demographic variables, health‐related factors and use of technology as potential predictors of health e‐mavenism through ordered regression analysis.

**Results:**

Confirmatory factor analyses showed that a second‐order two‐factor structure best captured the health e‐maven construct. Health e‐mavenism comprised two second‐order factors, each encompassing two first‐order dimensions: information acquisition (consisting of information tracking and consulting) and information transmission (consisting of information posting and sharing). Both first‐order and second‐order factors exhibited good reliabilities. Several factors were found to be significant predictors of health e‐mavenism.

**Discussion and conclusion:**

This study offers a starting point for further inquiries about health e‐mavens. It is a fruitful construct for health promotion research in the age of new media technologies. We conclude with specific recommendations to further develop the health e‐maven concept through continued empirical research.

## Introduction

Health information is increasingly digitalized. Mirroring Web 2.0, Health 2.0 is an expanding participatory, collaborative movement that transforms the traditional health‐care model through the use of diverse web‐based tools. The increasing adoption of ‘new health information technologies (HITs)’^1^ and web‐based health interventions^2^, ^3^, ^4^ is reshaping modes of health‐care delivery. Social media sites, such as YouTube, Facebook and Twitter, greatly increase the speed and scale of health information exchange with vast capacities for building and sharing user‐generated content.^5^ Using social media marketing to tailor messages and empower audiences is becoming a key strategy for health promotion programmes.^6^ This shift is reflected in the national objectives of *Healthy People 2020*, which include increasing engagement with health information through web‐based technologies and improving the quality of online health information.^7^ To help guide researchers in the study of individuals engaged in this new online health information trajectory, this study proposes the concept of ‘health e‐maven’ and empirically examines it with the Health Tracking Survey data from the Pew Internet & American Life Project.^8^

As health information becomes more consumer‐centred through web‐based technologies, it has become important to understand more about the individuals who are engaged in online health information activities. Existing efforts have emphasized different characteristics of online information users. For example, the construct *e‐patient* refers to an individual seeking health information online.^9^ *Lay information mediary* refers to ‘those who seek information on behalf or because of others’.^10^ *Health information gatekeepers* are defined as those in a position of authority, and hence control, over information access and flow (such as public or school librarians).^11^ Whereas these concepts advance different understandings of health information users, they are limited in capturing the group of active users who both seek and generate health‐related content online, functioning as an integral part of the consumer‐centred ‘peer‐to‐peer health care’.^12^ As such peer‐to‐peer health care increasingly supplements health care by specialists, these individuals are important actors for public health practitioners to identify and mobilize. A better understanding of these individuals is an important step towards harnessing their potential influence in a more informed and constructive way.

Drawing on the concept of the *maven* from the marketing literature, we name these individuals as *health e‐mavens*. Mavens are individuals with a general interest in a topic area who actively participate in information exchanges. The term ‘maven’ has been utilized to identify such individuals in a variety of domains. ‘Market mavens’ refer to the group of consumers who are ‘general market influencers’ actively involved in marketplace activities.^13^ They enjoy engaging in information exchanges across different products and are driven by a sense of pleasure or obligation to share information with others.^14^ The expanding online information activities and the growing importance of electronic word‐of‐mouth prompted the development of the ‘market e‐mavens’ concept^15^ (or ‘Internet mavens’^16^). Market e‐mavens, different from general market mavens, are ‘defined by the channel (i.e. email and the Internet) through which information is acquired and spread’.^15^ They are engaged specifically with the web space to accomplish their communication goals and are capable of searching for information online and responding to others’ information requests.^15^, ^17^ Identifying such market e‐mavens, for marketers, is of practical importance to the success of online viral campaigns.^15^

In health communication research, there is a general lack of attention to interpersonal influences in disseminating information and influence. Recently, concepts like ‘health maven’^18^ and ‘health information maven’^19^ have been developed to represent individuals who self‐identify as expert and influential information sources with respect to health issues. These concepts are not specifically designed to understand users of online health information. Parallel to the move from market maven to market e‐maven in the marketing literature, we propose the concept of *health e‐maven* through which to study individuals who are actively involved in *online* health information exchanges. Our conceptualization of health e‐mavens focuses on behavioural engagement in information activities. Congruent with the definition of market e‐mavens as ‘people who acquire and spread information via electronic platforms’,^15^ we define *health e‐mavens* as individuals who are consistently and actively involved with health information *acquisition* and information *transmission* on the web space. In general terms, information acquisition refers to processes of selecting, monitoring, filtering and collecting health information from various online sources, and information transmission refers to processes of spreading acquired knowledge, upon request or not, that aim to benefit other users.

For the health e‐maven construct to be useful in differentiating and understanding health information users, an empirical validation of the construct is needed. For this purpose, we use the Health Tracking Survey data from the Pew Internet & American Life Project.^8^ Reflecting the growing importance of online health information, the 2010 Health Tracking Survey includes four sets of questions on online health information seeking and sharing behaviours using web‐based technologies and social media platforms. These questions include online health information *tracking* (tracking health information on various devices), *consulting* (checking online ranking or reviews of doctors, hospitals or drugs), *posting* (posting online rankings or reviews of doctors, hospitals and drugs) and *sharing* (sharing health information on social media platforms). These questions represent a spectrum of popular online health information behaviours. They are apt measures for an initial empirical validation and examination of the health e‐maven construct by capturing both online health information *acquisition* (i.e. questions on information tracking and consulting) and *transmission* (i.e. questions on information posting and sharing), two aspects key to our conceptualization of the construct.

## Objectives

We aim to increase the understanding of active users of online health information via the health e‐maven concept through three specific objectives:

1. To establish the factor structure of the health e‐maven construct in order to empirically identify health e‐mavens. Specifically, we test the empirical viability of a second‐order two‐factor structure where information acquisition and information transmission are two second‐order factors, each consisting of two first‐order factors (online health information tracking and consulting, and online health information posting and sharing, respectively).
2. To assess the reliability and validity of the health e‐maven construct.
3. To determine what factors, including socio‐demographic variables, health‐related factors and use of technology, are associated with becoming a health e‐maven.

## Methods

### Data source

Data used in this study came from the 2010 Health Tracking Survey from the Pew Internet & American Life Project, an authoritative data source on civic engagement, politics, health and other issues.^8^ The survey, with the approval of Institutional Review Board,^20^ was conducted from 9 August to 13 September 2010 by Princeton Survey Research Associates International. The survey included various questions about Internet use and health care. The sample was representative of all adults (age 18 and older) in the USA with access to a landline or cellular phone. A random digit dialling (RDD) methodology was used, and seven or more attempts were made to obtain an interview from a sampled telephone number. The analyses reported in this study involved respondents who completed all of the measures key to the objectives of this study (i.e. questions on online health information seeking and sharing behaviours, to be detailed below).

### Analysis plan and study variables

First, descriptive statistics were derived on some basic demographic variables. A comparison between the included and the excluded samples on these variables was conducted. Second, corresponding to the three objectives outlined earlier, three major sets of analyses were conducted using stata (version 12) (StataCorp LP, College Station, Texas, USA).^21^

#### Establishing factor structure through confirmatory factor analysis

##### Analytical strategy

Establishing the factor structure of health e‐mavenism involved testing three models in sequence: Model 1, a first‐order four‐factor model (with tracking, consulting, posting and sharing being the four first‐order factors); Model 2, a second‐order two‐factor model (information acquisition consisting of tracking and consulting, and information transmission comprising posting and sharing, as specified in Objective 1); and Model 3, a second‐order one‐factor model (with the four first‐order factors loaded onto one singular second‐order factor). Model 1 was first examined for evidence of satisfactory model fit as well as reasonable factor loadings and interfactor correlations. These pieces of evidence, showing that the first‐order factors well explained the correlations among indicator items, were prerequisites for proceeding to a second‐order model.^22^ Given sufficient evidence from Model 1, Model 2 was examined for a more parsimonious account of the data. If its model fit was as good as or better than that of Model 1, the factor loadings were consistent and high, and second‐order factors explained a good amount of variance of the first‐order factors, Model 2 would be deemed as adequately accounting for the correlational structure of first‐order factors. In case of a high correlation between the two second‐order factors in Model 2, thus suggesting that the two factors might not be empirically distinguishable, Model 3 would be examined as a model with only one second‐order factor, the most parsimonious model. The final model was selected based on both model fit and parsimony.

These three models were sequentially estimated via confirmatory factor analyses (CFA) using the ‘sem’ package in stata 12.0 and evaluated based on two sets of criteria. First, following Kline's^23^ suggestions, we assessed the fit of each model using the following indices: (i) the model chi‐square, (ii) the Steiger–Lind root mean square of approximation (RMSEA, an index sensitive to the number of estimated parameters in the model),^24^ (iii) the Bentler comparative fit index (CFI, one of the indices least affected by sample size)^25^ and (iv) the standardized root mean square residual (SRMR, capturing the difference between observed and predicted correlations).^23^ For a model to be accepted, an RMSEA of 0.06 (or lower), a CFI of 0.90 (or higher, preferably 0.95) and an SRMR of 0.08 (or lower) are recommended.^26^ Second, we computed Bayesian information criteria (BIC)^27^, ^28^ for each model and calculated the difference in BIC values between models to compare models against each other. A negative value indicated good model fit, and a positive value suggested a problem with the model and difference in BIC values was interpreted as follows: a difference of two provided some evidence of model fit difference, six indicated strong evidence, and 10 or higher indicated very strong evidence.^27^

##### Variables used in CFA

The following four sets of questions from the survey were used in estimating the models. All of these questions were coded as 1 = ‘yes’ and 0 = ‘no’. ‘Don't know’ and ‘refused to answer’ responses were recoded as missing values. Specific wordings of all these questions are included in the Appendix. Health information tracking included a set of questions on whether respondents had performed a list of six behaviours of health information tracking such as ‘signed up to receive email updates or alerts about health or medical issues’ or ‘watched an online video about health or medical issues’. *Health information consulting* included three questions about whether respondents had engaged in online behaviours such as ‘consulted online rankings or reviews of doctors or other providers’. *Health information posting* included three questions on whether respondents had posted information about health‐care providers online such as ‘posted a review online of a doctor’. *Health information sharing* included questions on whether respondents had shared their health or medical experiences on a list of five online platforms such as ‘on a social networking site such as Facebook, MySpace or LinkedIn’ or ‘on Twitter or another status update site’.

#### Scale validation: reliability and construct validity

##### Analytical strategy

After CFA, we assessed the reliability and construct validity to further validate the scale. Reliability refers to the consistency of measurement. Although Cronbach's alpha is a popular coefficient of reliability, it has been shown to over‐ or underestimate the scale reliability of multi‐item measures under different circumstances.^29^ Raykov's scale reliability (ρ), developed based on the CFA measurement model, is a direct measure of a scale's true‐score variance relative to total observed variance and resolves the issues of Cronbach's alpha. We calculated Raykov's scale reliability coefficients for each factor.

To further establish construct validity, correlation patterns between factors and several external variables were examined. If the derived factor structure was valid, an external variable should correlate to a similar degree with the pair of first‐order factors loaded on the same second‐order factor, and correlate differently with the two distinguishable, second‐order factors. Pearson correlation coefficients (*r*) were computed to represent the direction and strength between two variables. As the correlation coefficients under comparison were from the same sample, statistical differences between correlation coefficients were tested using Meng, Rosenthal & Rubin's^30^ *z*‐score, a test specifically designed to compare two or more dependent correlation coefficients.

##### Variables used in scale validation

The following external variables available in the data set were used. *Positive experiences with online health information* asked the respondents, ‘have you or has anyone you know been helped by following medical advice or health information found on the Internet?’ (reversely coded as 1 = ‘no’, 2 = ‘minor help’, 3 = ‘moderate help’ and 4 = ‘major help’). *Negative experiences with online health information* were measured by asking respondents, ‘have you or has anyone you know been harmed by following medical advice or health information found on the Internet?’ (reversely coded as 1 = ‘no’, 2 = ‘minor harm’, 3 = ‘moderate harm’ and 4 = ‘major harm’). *The variety of health information sought* asked whether they had ‘ever looked online for’ a list of 15 health‐related issues (1 = ‘yes’ and 0 = ‘no’), such as ‘a specific disease or medical problem’, ‘a certain medical treatment or procedure’ or ‘doctors or other health professionals’. An additive index of these items was created to represent the total number of health issues for which an individual sought information online.

#### Predicting health e‐mavenism via ordered logistic regression

##### Analytical strategy

Based on the confirmed factor structure, we constructed an ordinal variable of health e‐mavenism (‘inactive users’, ‘active users’ and ‘health e‐mavens’). An ordered logistic regression analysis, designed to estimate the relationship between independent variables and an ordinal dependent variable, was run via the command ‘ologit’ in stata 12.0 to examine how a host of predictors related to health e‐mavenism. An important assumption of logistic regression, the parallel regression assumption, was first tested. This assumption states that the relationship between a predictor and the ordinal outcome variable is parallel across all levels of the outcome variable. Logit coefficients, analogous to beta coefficients for multiple regression models, were presented corresponding to each predictor variable. Odds ratios, which translated logit coefficients to the odds of being in a higher level of the outcome variable associated with a unit change of a predictor variable, were also computed to ease interpretations.

##### Variables used in ordinal regression

In addition to demographic variables, the following variables were included as predictors in the regression model. *Health conditions* was an additive index based on responses to six questions asking whether the respondent was ‘now living with any of the following health problems or conditions: diabetes, high blood pressure, asthma or other lung conditions, heart disease, cancer or any other chronic health problems’. *Health insurance coverage* was an additive index of health insurance coverage based on five questions about insurance (‘Are you now personally covered by… private health insurance offered through an employer or union, that you bought yourself, Medicaid, Medicare, or other health insurance including military or veteran coverage?’). *Frequency of Internet use* was assessed by averaging the responses to two questions ‘how often do you use the Internet or email from home/work?’ (0 = ‘never’, 6 = ‘several times a day’).

## Results

### Demographics and descriptive statistics

The sample size for the survey was 3001, but the number of individuals included in this study was 402 due to missing data on key variables used in factor analysis. Our sample of 402 respondents consisted of 62.4% females and 37.6% males (Table 1). The majority of these respondents were White (66.1%), followed by Black or African American (23.2%); only a small portion (15.6%) reported being of Hispanic origin. Twenty‐two respondents did not finish high school (5.5%), 222 received a high school degree or some vocational school or college training (55.2%), and 156 had a college degree or higher (38.8%). The median annual household income fell within the $50 000–$75 000 range, and 39.5% of respondents reported an annual household income below $40 000. Respondents’ ages ranged from 18 to 88 years (M* *=* *33.88, SD = 13.54, not reported in the table).

**Table 1:** Demographic characteristics of the included sample compared to the excluded sample

Demographic characteristics	Included (% of 402)	Excluded (% of 2599)	Sample comparison
Sex			
Male	37.6	41.1	χ^2^(1) = 1.84
Female	62.4	58.9	*P *=* *0.175
Race			
White	66.1	67.4	
Black or African American	23.2	25.0	
Asian or Pacific Islander	5.1	2.2	
Mixed race	2.6	2.1	
Native American/American Indian	1.0	1.2	
Other	2.0	2.1	*P *=* *0.067^a^
Ethnicity			
Hispanic	15.6	16.7	χ^2^(1) = 0.31
Not Hispanic	84.4	83.3	*P *=* *0.574
Family income of 2009			
Less than 20 000	19.2	29.2	
20 000–40 000	20.3	25.4	
40 000–75 000	29.7	23.0	χ^2^(3) = 29.18
75 000 or higher	30.8	22.4	*P *=* *0.000
Education			
Not finish high school	5.5	15.7	
High school degree	25.3	32.3	
Some vocational school or college	29.9	22.8	
College degree or higher	38.8	28.6	
Do not Know/Refuse	0.5	0.6	*P *=* *0.000^a^
Age group			
18–24 years	30.6	9.1	
25–44 years	48.2	24.5	
45–64 years	18.4	38.8	
65–95 years	2.8	27.5	
Do not Know/Refuse	0	0.1	*P *=* *0.000^a^

a Fisher's exact test was used for variables where at least one cell had an expected frequency of five or less, which violates the assumption of the chi‐square test. Fisher's exact test has no such assumption and thus was used as the appropriate test for these cases. Fisher's exact test does not have a test statistic and only yields the *P*‐value.

A comparison of respondents included in this sample (*n *=* *402) and those excluded (*n *=* *2599) showed no difference in terms of gender, race or ethnicity. Our sample, however, had significantly higher levels of education and higher family income and were younger in age (Table 1).

### Factor structure of health e‐mavenism (objective 1)

#### Confirmatory factor analyses

Three confirmatory factor analyses were performed to assess the factor structure of health e‐mavenism. Model fit indices for these three models are presented in Table 2. Model 1, the first‐order four‐factor model, provided a good fit to the data, with RMSEA below 0.06, CFI above 0.90 and SRMR below 0.08 (the first row of Table 2). Factor loadings were consistent and reasonably high (from 0.40 to 0.76, not shown in the table). Furthermore, interfactor correlations (not shown in the table) were substantial (from 0.45 to 0.80), especially between tracking and consulting, which were theorized to capture ‘information acquisition’ (0.81), and between posting and sharing, which were intended to measure ‘information transmission’ (0.59). These pieces of evidence all attested to the empirical feasibility of a higher‐order model.

**Table 2:** Confirmatory factor analysis models: model fit and comparisons

Model	Description	χ^*2*^	d.f.	RMSEA	CFI	SRMR	BIC^b^
1	First‐order four‐factor	264.49	111	0.059 *P *=* *0.058^a^	0.915	0.046	−401.11
2	Second‐order two‐factor	271.53	112	0.060 *P* = 0.041^a^	0.912	0.047	−400.10
3	Second‐order one‐factor	285.95	113	0.062 *P *=* *0.016^a^	0.904	0.053	−392.60

*N* = 402. RMSEA, root mean square of approximation; CFI, comparative fit index; SRMR, standardized root mean square residual; BIC, Bayesian information criterion, computed as χ^*2*^ – Ln(N)* d.f. where χ^*2*^ is the minimum function chi‐square.

a *P‐*value refers to the probability that RMSEA ≤0.05.

b Model 2 vs. Model 1: BIC difference = 1.01; Model 3 vs. Model 2: BIC difference = 7.50.

**Figure 1:**
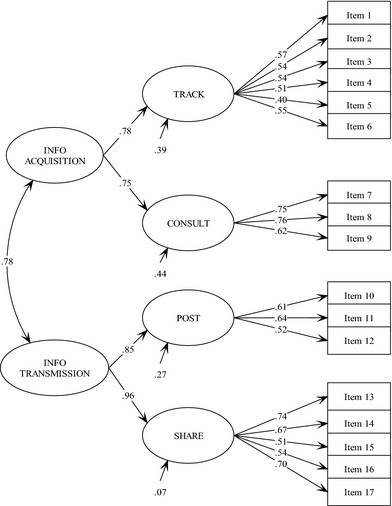
Standardized Parameter Estimates of the Second‐Order Two‐Factor Model (Model 2). Notes. Second‐order factors: Online health information acquisition and Information transmission. First‐order factors: Online health information tracking, consulting, posting and sharing. *N* = 402, χ^2^(112)* = *271.53 (*P *<* *0.001), RMSEA = 0.060 with 90% confidence interval [0.051, 0.069], CFI = 0.91, SRMR = 0.047 (RMSEA, root mean square of approximation; CFI, comparative fit index; SRMR, standardized root mean square residual). Specific item wordings are included in the Appendix.

We then proceeded to test Model 2, the second‐order two‐factor model, in which tracking/consulting and posting/sharing were loaded onto their respective second‐order factors (as depicted in Fig. 1). As shown in the middle row of Table 2, Model 2 yielded satisfactory fit indices that were very close to those of Model 1. The BIC difference between the two models (1.01) indicated that Model 2 provided a solution as equally good fitting as Model 1. As shown in Fig. 1, the first‐order factors had high and parallel factor loadings on their respective second‐order factors (0.78 and 0.75 for tracking and consulting on ‘information acquisition’, 0.85 and 0.96 for posting and sharing on ‘information transmission’). The second‐order factors accounted for 56–93% of the variances in the first‐order factors (not reported in the table or figure; they were derived as ‘1 – error variance’, with the error variance for each of the four first‐order factors depicted in Fig. 1). Model 2, therefore, provided an adequate account for the correlational structure among the first‐order factors.

As the two second‐order factors in Model 2 were highly correlated at 0.78 (Fig. 1), we investigated the possibility that the four first‐order factors would load on only one second‐order factor (Model 3). The bottom row of Table 2 showed that model fit indices for Model 3 were only slightly worse than Model 2 with higher RMSEA, lower CFI and higher SRMR. BIC difference between Model 3 and Model 2 (7.50), however, equated to strong evidence of a worse fit for Model 3.^27^ In other words, Model 3, although more parsimonious than Model 2, resulted in a substantial decrease in model fit.

Therefore, on account of both model fit and parsimony, we selected Model 2 as the optimal factor structure to describe the construct of health e‐mavenism. In other words, health e‐mavenism should be measured with respect to two dimensions, information acquisition and information transmission, each in turn measured by two sets of indicators (online information tracking and consulting, and online information posting and sharing).

### Scale validation (objective 2)

#### Assessing reliability

Raykov's scale reliabilities for the four first‐order factors ranged from 0.87 to 0.96 (the last column in Table 3), indicating high internal consistency among the items for each first‐order factor. The reliabilities for the second‐order factors were also satisfactory: 0.73 for information acquisition and 0.93 for information transmission.

**Table 3:** Examining construct validity: correlations between factors and external variables

Factors	Positive experience	Negative experience	Info‐seeking variety	ρ
First‐order factors				
Track	0.42***	0.04	0.63***	0.87
Consult	0.32***	0.07**	0.58***	0.91
Share	0.22***	0.13***	0.32***	0.92
Post	0.15***	0.13***	0.25***	0.96
Second‐order factors				
Acquisition	0.43***	0.05*	0.67***	0.73
Transmission	0.23***	0.14***	0.33***	0.93

*N* = 402. *P <* *0.05, **P <* *0.01, ***P <* *0.001.

Cell numbers are correlation coefficients except for the last column which contains scale reliability coefficients for each factor.

Raykov's scale reliability is calculated using Equation 8.2 in Brown.^22^

$\rho =\;{\left(\sigma \lambda_{i}\right)}^{2}/\left[{\left(\sigma \lambda_{i}\right)}^{2}+\sigma \theta_{ii}\right],$

where (Σλ_1_)^2^ is the squared sum of unstandardized factor loadings, and Σθ_*ii*_ is the sum of unstandardized measurement error variances.

#### Assessing construct validity

We used three external variables available in the data set to further examine the construct validity: *positive experiences with online health information* (‘no help’ = 54.0%, ‘minor help’ = 13.5%, ‘moderate help’ = 21.6% and ‘major help’ = 10.9%), *negative experiences with online information* (‘no harm’ = 96.2%, ‘minor harm’ = 2.3%, ‘moderate harm’ = 0.7% and ‘major harm’ = 0.8%) and *variety of health information sought online* (range: 0–15, *M *=* *4.25, SD = 3.64).

Table 3 presents the correlations of these external variables with the first‐order factors and with the second‐order factors. As previously mentioned, these external variables were expected to correlate similarly with each pair of first‐order factors loaded on the same second‐order factor and correlate differently with the two second‐order factors. Using Meng *et al*. (1992)'s^30^ *z‐*test (*z‐*scores not reported in the table), the patterns of correlations supported these expectations. On the one hand, all three external variables had statistically comparable correlations with each pair of first‐order factors loaded on the same higher‐order factor. On the other hand, they had significantly different correlations with the two second‐order factors. To be more specific, positive experiences correlated with the information acquisition factor to a stronger degree than with the transmission factor (*z* = 6.43, *P *<* *0.001). Negative experiences, on the contrary, had a higher correlation with the information transmission factor than with the acquisition factor (*z* = 2.72, *P *<* *0.01). These findings suggested that an individual who had benefited from online information before would be more likely to engage in further online information acquisition behaviours and that someone who had been harmed by online information before would be more likely to engage in more transmission behaviours. The variety of information sought was related more strongly with the information acquisition factor than with the transmission factor (*z *=* *12.23, *P *<* *0.001).

### Predicting health e‐mavenism (objective 3)

Based on the second‐order two‐factor model, we constructed variables for *information acquisition* by adding up reported incidences of online information tracking and consulting (0 = 24.1, 1 = 16.9, 2 = 13.2, 3 = 10.1, 4 = 11.2, 5 = 8.5, 6 = 7.2, 7 = 3.5, 8 = 3.7, 9 = 0.8%) and *information transmission* as the sum of online information posting and sharing (0 = 69.2, 1 = 13.7, 2 = 7.0, 3 = 2.7, 4 = 2.5, 5 = 2.2, 6 = 1.2, 7 = 0.8, 8 = 0.8%). We then dichotomized each of these two variables into high and low categories. Those who were low on both dimensions were regarded as inactive information users (*n *=* *188, 46.8%); those who were high on only one dimension were active information users (*n *=* *120, 29.8%); and those who were high on both dimensions were categorized as health e‐mavens (*n *=* *94, 23.4%). These three groups, categorical and ordered, formed an ordinal scale that represented the propensity towards being a health e‐maven.

Using this ordinal scale as the dependent variable, we ran an ordered logistic regression model to find out what factors predicted health e‐mavenism. These predictors included socio‐demographic factors (i.e. educational level, gender, race and ethnicity), health‐related factors (health conditions, range: 0–5, M* *=* *0.49, SD = 0.86; and health insurance coverage, range: 0–4, M* *=* *1.18, SD = 0.75), as well as Internet‐use frequency (based on measures of Internet use at home/at work: ‘never’ = 4.2%/38.2%, ‘less often’ = 1.7%/3.3%, ‘every few weeks’ = 1.2%/0.2%, ‘1–2 days a week’ = 3.5/2.8%, ‘3–5 days a week’ = 11.2%/4.7%, ‘about once a day’ = 17.9%/9.3% and ‘several times a day’ = 60.2%/41.5%; for the averaged scale, range: 0–7, M* *=* *4.18, SD = 1.72).

The test of the parallelism assumption was non‐significant (χ^*2*^ = 5.67, d.f. = 12, *P *=* *0.93), indicating that the assumption was not violated. In other words, an independent variable had the same effect on the probability of moving from the inactive user group to the active user group and e‐maven group combined, and the probability of moving from the combined inactive and active user group to the e‐maven group. Logit coefficients and odds ratios (OR) are shown in Table 4.

**Table 4:** Generalized ordered logit estimates for ordinal regression model

	Logit (SE)	Odds ratio (SE)
Demographics		
Gender (Reference category: male)	0.42* (0.21)	1.52* (0.32)
Age	−0.00 (0.01)	0.99 (0.01)
Education	0.15* (0.07)	1.16* (0.89)
Race (Reference category: White)		
African American	−0.34 (0.25)	0.71 (0.18)
Asian or pacific islander	−1.10* (0.49)	0.33* (0.16)
Mixed race	−0.01 (0.61)	0.99 (0.60)
Native american	−14.22 (487.63)	0.00 (0.00)
Other	−2.27* (1.12)	0.10* (0.11)
Ethnicity (Reference category: of Hispanic/Latino origin)	−0.27 (0.29)	0.76 (0.22)
Health‐related factors		
Health conditions	0.41*** (0.13)	1.50*** (0.19)
Health insurance coverage	0.33* (0.16)	1.39* (0.22)
Internet use		
Internet‐use frequency	0.14* (0.07)	1.15* (0.08)

SE, ‘Standard errors’. *P <* *0.05, ***P <* *0.001.

*N* = 383. Dependent variable: Health e‐mavenism was constructed as −1 = ‘inactive users’, 0 = ‘active users’ and 1 = ‘health e‐mavens’.

Among the demographic variables, gender, education and race were significantly associated with health e‐mavenism. Being female and having a higher level of education were translated to, respectively, a 52% and 16% greater likelihood that one would be in a higher category on the e‐maven scale (OR = 1.52, *P *<* *0.05, and 1.16, *P *<* *0.05, respectively). Being Asian or Pacific Islander, or being in the ‘other’ race groups, was negatively related to health e‐mavenism, meaning that one had a 67% (OR = 0.33, *P *<* *0.05) or 90% (OR = 0.10, *P *<* *0.05) greater likelihood of being in a lower category on the e‐maven spectrum (i.e. a less active information user) compared to Caucasians. Having more chronic health conditions was also related to greater online health information activity: an increase of one chronic problem faced by an individual was associated with a 50% greater likelihood that he/she became a more active user of online health information (OR = 1.50, *P *<* *0.001). Health insurance plans (OR = 1.39, *P *<* *0.05) and Internet‐use frequency (OR = 1.15, *P *<* *0.05) also positively predicted the likelihood of becoming more engaged in online health information activities.

## Discussion

The changing landscape of media use has rendered online health information activities increasingly important to health education and promotion efforts. This paper analysed online health information users through the concept of ‘health e‐maven’ and empirically validated its behavioural dimensions. Confirmatory factor analyses showed health e‐mavenism to consist of two primary facets of online behaviours: online health information acquisition and transmission. In other words, health e‐mavens are individuals who can be identified and characterized by both information seeking and sharing behaviours. Health information acquisition consisted of two specific activities, health information tracking and consulting, and health information transmission comprised information sharing and online posting activities. The ordered regression revealed that several factors were associated with health e‐mavenism. Individuals who were female or had a higher education level were more likely to be health e‐mavens; racial minorities were less likely to engage health information acquisition and transmission activities. These results are consistent with previous findings on the characteristics of online information users.^31^ Both health status and health insurance were positive predictors of health e‐mavenism, suggesting that individuals with more health problems and more health insurance plans were more likely to become active users of online information. As health e‐mavenism is a channel‐specific concept, frequency of Internet use was also a positive predictor.

The health e‐maven concept builds on previous literatures on market mavens, market e‐mavens and health mavens. It is a fruitful concept for health communication scholars to further develop, with promises for theoretical and practical insights for health promotion research in the age of new and social media technologies. Theoretically, interpersonal influence has been gaining greater importance as the mass media becomes increasingly individuated, and network connections, online or offline, constitute a vital force to generate or facilitate social influence. The health e‐maven concept presumes and amplifies the importance attached to interpersonal connections and can potentially elucidate processes of diffusion through online networks. Practically, with the exponential growth of Internet capacities, especially the popularity of social networking sites in the past decade, those who are interested in health information and helping others can now extend their influences from local communities to the virtual space. Health e‐mavens serve as a liaison between the large stock of health information online and others who are less engaged or less competent users. They can also simplify and translate medical terms or jargon that may otherwise be difficult for people of relatively low health literacy. Health e‐mavens can thus be a critical group for health promotion practitioners to recruit and mobilize in various web‐based health intervention programmes or online health communication campaigns.

As the first to propose and examine this concept, our study is obviously limited in several ways. First and foremost, the secondary data used in the analyses were constraining. Our analysis involved a reduced sample size due to missing data. Individuals of lower education and income were underrepresented. This bias in our sample may in itself bespeak the digital disparities in access to online health care. Future efforts should be made to study these groups specifically in terms of their use of online health information. In addition, the questions used in confirmatory factor analysis were dichotomous measures. Future studies should develop ordinal or interval measures and collect primary data to better capture the underlying continuum of these behaviours.

The secondary data also prevented us from exploring more interesting theoretical relationships. We could not examine the effects of some variables of interest, such as trust of online health information (particularly regarding user‐generated content), social network size and health literacy, or the actual or perceived influence of health e‐mavens. Future studies should develop ‘a nomological network’^32^ that encompasses theoretically derived relationships in order to map out the theoretical role of health e‐mavens in the larger process. In addition, future research should also examine the relationship between health mavens and health e‐mavens. One basic question is the extent to which and/or under what conditions these two constructs differ from each other. Other important questions abound. For example, what may be different causes and effects associated with each construct? In what kind of health‐care delivery or health promotion settings can one construct be more applicable and productive than the other? How can the two work in conjunction to best advance health promotion efforts? Finally, future research should continue to differentiate health e‐mavens from other types of active online users of health information. For example, health e‐mavens are defined by engagement in both seeking and sharing information, thus serving as digital hubs for health information across their social networks, while other active online users may limit activity to only one of these behavioural domains. These questions require further theoretical development and empirical examination in future studies.

Despite these limitations, health e‐maven is a promising concept for health communication research and practice. This study offers a starting point for further conceptual thinking and empirical inquiries. With its potential to become a productive concept at the crossroads between interpersonal communication, health campaign research, Internet research and social network analysis, we believe the health e‐maven concept will elicit interest from different areas of communication and health research.

## Appendix

Items and dimensions of the health e‐maven construct

**Table:**

Dimensions	Items
Tracking	
1	Have you signed up to receive email updates or alerts about health or medical issues?
2	Have you read someone else's commentary or experience about health or medical issues on an online news group, website or blog?
3	Have you watched an online video about health or medical issues?
4	Have you gone online to find others who might have health concerns similar to yours?
5	Have you tracked your weight, diet or exercise routine online?
6	Have you tracked any other health indicators or symptoms online?
Consulting	
7	Have you consulted online rankings or reviews of doctors or other providers?
8	Have you consulted online rankings or reviews of hospitals or other medical facilities?
9	Have you consulted online reviews of particular drugs or medical treatments?
Posting	
10	Have you posted a review online of a doctor?
11	Have you posted a review online of a hospital?
12	Have you posted your experiences with a particular drug or medical treatment online?
Sharing	
13	Have you posted about health or medical issues in an online discussion, a listserv, or other online group forum?
14	Have you posted about health or medical issues on a social networking site such as Facebook, MySpace or LinkedIn?
15	Have you posted about health or medical issues on a blog?
16	Have you posted about health or medical issues on Twitter or another status update site?
17	Have you posted about health or medical issues on a website of any kind, such as a health site or news site that allows comments and discussion?
